# Severe stunting and its associated factors among children aged 6–59 months in Ethiopia; multilevel ordinal logistic regression model

**DOI:** 10.1186/s13052-021-01110-8

**Published:** 2021-07-26

**Authors:** Amare Muche, Reta Dewau

**Affiliations:** School of Public Health, College of Medicine and Health Sciences, Wollo University, Dessie, Ethiopia

**Keywords:** Stunting, Severe stunting, Children, Ordinal logistic regression, Ethiopian demographic and Health Survey (EDHS), Ethiopia

## Abstract

**Background:**

In Ethiopia, stunting is the most common form of undernutriton. Identifying the determinants of severe stunting among children is crucial for public health interventions to improve child health. Therefore, this study aimed to identify the determinants of severe stunting among under-five children in Ethiopia.

**Methods:**

A community-based cross-sectional study design was employed. A two stage stratified cluster sampling technique was used. A multilevel ordinal logistic regression model was fitted to identify independent determinants. Adjusted odds ratio (AOR) and median odds ratio (MOR) with its 95% confidence interval at *p*-value< 0.05 were used to declare statistical significance.

**Results:**

The result of this study showed that about 18% of the children were severely stunted. Being male increased the severity of stunting in children by 26% adjusted odds ratio (AOR): 1.26 (95% CI: 1.09–1.46), compared to female sex; over-weight mothers increased the severity of stunting in their children AOR: 3.43 (95% CI: 2.21–5.33) compared to normal BMI mothers; and children from middle, poorer, and poorest wealth index households were 1.84 (95% CI:1.27–2.67), 2.13 (95% CI, CI:1.45–3.14) and 2.52 (95% CI,1.72–3.68). In contrast, severe stunting was reduced by 62% (AOR: 0.38, 95% CI: 0.20–0.74) and 48% (AOR = 0.52, 95% CI: 0.37–0.72) in children of educated mothers compared to children of uneducated mothers and children of underweight mothers compared with those children of normal BMI mothers respectively. For each one-unit increase in maternal height, there is a 5% significant reduction in the child’s odds of being severely stunted. After controlling for other factors, the effect of predictors on the likelihood of stunting in high risk clusters increased by a median odds ratio (MOR) of 1.83 (95% CI: 1.69–2.00).

**Conclusions:**

The magnitude of severe childhood stunting was still high with regional variation in Ethiopia. Child age, sex, maternal height, age, education and household wealth index as well as administrative regions were significantly associated factors with severe stunting. Significant interventions shall be implemented at the individual, household and community levels in order to reduce the problem.

## Background

Linear growth faltering in childhood/stunting is the common condition of malnourished children, affecting an estimated 161 million children worldwide in 2013 [1]. More than half (55%) of all stunted children under 5 lived in Asia and more than one third (39%) lived in Africa in 2018 [2]. Currently, in Ethiopia the prevalence is estimated to be about 38% among children from 0 to 59 months [3].

Stunting is less than minus two (− 2) standard deviation (SD) from the length/height for age World Health Organization Child Growth Standards median. While severe stunting is less than minus three (− 3) SD from the length/height for age World Health Organization Child Growth Standards median [4]. Stunted children suffer from irreversible physical and cognitive damage as a result of their stunted growth. Also the consequence of childhood stunting might last lifelong in adulthood and even pass to the next generation [1, 5].

Child stunting can happen in the first 1000 days of life after conception and is related to many different factors which include socio-demographic, dietary intake, infections, maternal nutritional status, infectious diseases, and micronutrients deficiencies, and the environment [6, 7]. Children who are stunted due to iodine and iron deficiencies may suffer from irreversible brain damage, preventing them from reaching their full developmental potential. They also have a shorter adult height and higher susceptibility to chronic disease in adulthood, lower attained schooling, and reduced adult income [8]. Stunted children also have a higher mortality risk than non-stunted children [9].

Previously, various studies revealed that child’s age [7, 10–16], child’s sex [7, 10, 11, 15–18], type of birth [7, 18] previous birth interval [7], child have diarrhea [7, 19], number of family members in the household [7], maternal educational level [10, 14–16, 18, 19], fathers’ educational level [7, 12, 18], wealth index [7, 10, 11, 14, 15, 18, 20] source of drinking water [15, 16], type toilet facility [7, 16, 18], maternal BMI [7, 18], maternal height [7, 14], residence [14], region [18] were independent predictors of severe stunting in children.

Different policies, programs and strategies are being implemented in the world to reduce the problem of childhood stunting as well in Ethiopia. The Global Nutrition Targets 2025 [21], a 40% reduction in the number of children under-5 who are stunted, as endorsed by the sixty-fifth World Health Organization Assembly in 2012 [22, 23]. Ethiopia is implementing national Nutrition Programs phase I (2010–2015) and phase II (2016–2020) targeted at the reduction of childhood stunting of children aged under-five [24]. In addition, strategies like Infant, Young Children, Adolescents and Maternal Nutrition are aimed to improve this inter-generational cycle of childhood stunting in the country [25, 26].

Despite these interventions, the magnitude of stunting in children is still high in Ethiopia. Moreover, previous studies were limited identifying factors associated with overall childhood stunting and these studies were limited to small areas to represent a national problem. Thus, the current study aimed to assess severe childhood stunting and its associated factors in children aged from 6 to 59 months in Ethiopia using national representative data.

## Methods and materials

### Study design and settings

Community-based cross-sectional study design was conducted among children aged 6–59 months. The 2016 Ethiopian Demographic and Health Survey (EDHS) is the fourth Demographic and Health Survey conducted in Ethiopia, which was conducted from January 18, 2016 to June 27, 2016 [3].

Administratively, Ethiopia is divided into nine geographical regions and two administrative cities. The sampling frame used for the 2016 EDHS is the Ethiopia Population and Housing Census (PHC), which was conducted in 2007 by the Ethiopian Central Statistical Agency. The census frame is a complete list of 84,915 enumeration areas (EAs) created for the 2007 PHC. An EA is a geographical area covering on average 181 households [3].

### Source population and study population

The source populations are children aged 6–59 months who were residing in the households in Ethiopia. The study population is children aged 6–59 months who were living in the selected households in Ethiopia.

### Inclusion and exclusion criteria

All children aged 6–59 months in the selected households. Children who fulfilled the inclusion criteria had severe medical conditions at the time of survey.

### Sampling procedure

In two phases, the 2016 EDHS sample was stratified and picked. There were 21 sampling strata in each region, which were divided into urban and rural areas. In two phases, EA samples were selected independently in each stratum. Implicit stratification and proportional allocation were achieved at each of the lower administrative levels by sorting the sampling frame within each sampling stratum before sample selection, according to administrative units in different units in different levels, and by using a probability to size selection at the first stage of sampling [3].

In the first stage, a total of 645 (202 in urban areas and 443 in rural areas) were selected with probabilities proportional to EA size (based on the 2007 PHC) and with independent selection in each sampling stratum. A household listing operation was carried out in all of the selected EAs from September to December 2015. The resulting lists of households served as a sampling frame for the selection of households in the second stage. Some of the selected EAs were large, consisting of more than 300 households. To minimize the task of household listing, each large EA selected for the 2016 EDHS was segmented. Only one segment was selected for the survey with probability proportional to segment size. Household listing was conducted only in the selected segment; that is, a 2016 EDHS cluster is either an EA or a segment of an EA of 2007 [3].

In the second stage of selection, a fixed number of 28 households per cluster were selected with an equal probability systematic selection from the newly created household listing. All women age 15–49 and all men age 15–59 who were either permanent residents of the selected households or visitors who stayed in the household the night before the survey were eligible to be interviewed. In all of the selected households, height and weight measurements were collected from children age 0–59 months. Anemia testing was performed on children age 6–59 months whose parent/guardian consented to the testing [3].

### Variables of the study

Dependent variable – Stunting (ordinal variable which was categorized as severely stunted if a child’s HAZ score less than − 3 SD, moderately stunted (− 3 ≤ HAZ < − 2), not stunted (HAZ ≥ − 2 SD) [4, 27].

Covariates – community level factors: region, enumeration areas (EAs/clusters), residence, community education and community wealth index.

Household factors: wealth index, sex of household head, family size, number of under five children in the household, source of drinking water and type of toilet facility.

Maternal characteristics: educational level, marital status, body mass index (BMI), maternal height, birth interval and paternal education.

Child characteristics: age, sex, type of birth, birth order and anemia level.

### Operational definitions

Improved drinking water source: include piped water, public tap, standpipes, tube wells, boreholes, protected dug wells and spring, rain water, and bottled water [28].

Unimproved drinking water source: include unprotected well, unprotected spring, surface water (river/dam/lake/pond/stream/canal/irrigation channel), tanker truck, cart with small tank and other [28].

Improved toilet facility: include any non-shared toilet of those types: flush/pour flush toilets to piped water systems, septic tanks and, and pit latrines; ventilated improved pit (VIP) latrines, pit latrines with slabs, and composting toilets [28].

Unimproved toilet facility: includes flush to somewhere else, flush do not know where, pit latrine without slab/open pit, no facility/bush/field, bucket toilet, hanging toilet/latrine, others [28].

Anemia level: hemoglobin levels are adjusted for altitude in enumeration areas that are above 1000 m. Not anemic (≥11.0), mild anemic (10.0–10.9), moderate anemic (7.0–9.9), severe anemic (< 7.0) [3].

Body mass index: BMI is calculated by divided weight in kilograms by height in meters square (kg/m^2^). Women aged 15–49 years who are not pregnant and who have not had a birth in the last 2 months before the survey. Underweight (BMI < 18.5 kg/m^2^), normal (BMI 18.5–24.9 kg/m^2^) and over weight (≥25.0 kg/m^2^) [3].

### Anthropometric measurements

The length of children aged < 24 months was measured during the survey in a recumbent position to the nearest 0.1 cm using a locally made measuring board (Shorr Board®) with an upright wooden base and moveable headpieces. Children ≥24 months were measured while standing upright. The length/height-for-age Z-score, an indicator of nutritional status, was compared with reference data from the WHO Multicenter Growth Reference Study Group, 2006 [29]. Children whose height-for-age Z-score is < − 2 SD from the median of the WHO reference population are considered stunted (short for their age).

### Data processing and analysis

The EDHS dataset accessed available at the DHS website; https://www.dhsprogram.com/data/available-datasets.cfm after registering for the dataset access permission the 2016 EDHS data set was accessed through the DHS website; https://dhsprogram.com/data/dataset_admin/login_main.cfm . The kid recode (KR) data set in STATA file was the data set containing the outcome and predictor variables of our study. The data was explored, cleaned, coded, re-categorized and recoded to be suitable for analysis.

This study was based on secondary data analysis of 2016 EDHS by adjusting sampling weights. Categorical characteristics and outcome of the study was described in terms of percentage and frequencies. Tables, bar graph and pie chart were used to present the data some selected variables which has significant association with stunting. A bi-variable multi-level ordinal logistic regression analysis was carried out to see the crude effect of each independent variable on stunting, and then variables with *p*. value of < 0.25 were entered to the multivariable multi-level ordinal logistic regression analysis.

The deviance information criterion (DIC) [30] statistic was calculated for the different models (individual level, community level and both individual and community level) fitted with logit, probit and clog log link functions. The DIC was used to evaluate and compare model performance of the full model and the reduced model. A model with lower DIC was considered as one with a better fit.

Variance partition coefficient (VPC) [31] median odds ratio (MOR) [31] and proportional change in variance (PCV) statistic were calculated to measure the variation between clusters (the random effect variable). VPC represents the percentage variance explained by higher level (clusters). Hence, it was calculated as below.

$\mathrm{VPC}=\frac{\upsigma_{\mathrm{u}}^2}{\left({\upsigma}_{\mathrm{e}}^2+{\upsigma}_{\mathrm{u}}^2\right)}$, where ${\upsigma}_{\mathrm{u}}^2$ is the between cluster (Enumeration area) variance, ${\upsigma}_{\mathrm{e}}^2=3.29$ [31, 32].

Median odds ratio is the median value of the odds ratio between the highest risk and the area at lowest risk when randomly picking out two areas and it was calculated using the formula; $\mathrm{MOR}=\exp \left(\sqrt{\left(2\ast {\upsigma}_{\mathrm{u}}^2\times 0.75\right)}\ \right)\approx \exp \left(0.95{\upsigma}_{\mathrm{u}}^2\right)$ [31, 33, 34].

The proportional change in variance (PCV) measures the total variation attributed by the individual level factors and area level in the multilevel model. The PCV is calculated as:

$\mathrm{PCV}=\frac{\left({\mathrm{V}}_{\mathrm{A}}-{\mathrm{V}}_{\mathrm{B}}\right)}{{\mathrm{V}}_{\mathrm{A}}}\times 100,$ Where V_A_ = variance of the initial Model, V_B_ = variance of the model with more terms [31, 33, 34].

### Ethical consideration

The data was downloaded after the purpose of the analysis was sent and access permission received confirmation letter that was approved by MEASURE DHS. The original data was collected in confirmation with international and national ethical guidelines. Ethical clearance for the survey was provided by the Ethiopian Public Health Institute (EPHI) Review Board, the National Research Ethics Review Committee (NRERC) at the Ministry of Science and Technology, the Institutional Review Board of ICF Macro International, and the United States Center for Disease Control and Prevention (CDC).

The Ethiopian Demographic and Health Survey ensured the principle of respondent’s protection and prevention from unnecessary risk. Verbal informed consent was obtained from participants before data collection began. Participants were informed of their anthropometric measurements (weight, height and edema screening).

## Result

### Socio-demographic characteristics of the study participants

A total of 8122 children aged from 0 to 59 months were included to this study. The median age of the study participants was 28 months with inter quartile range (IQR) =34 months. Likewise, the mean maternal height was 158 cm with its standard deviation (SD) =6.8 cm. Most of the study participants (51.53%) were male children (Table 1).

**Table 1:** Socio-demographic characteristics of the study participants, 2016 (*n* = 8122)

Variables	Frequency	Percentage (%)
Age of the child		
6–11 months	981	12.08
12–23 months	1798	22.14
24–35 months	1726	21.25
36–47 months	1752	21.57
48–59 months	1865	22.96
Sex of the child		
Male	4185	51.53
Female	3937	48.47
Mothers’ educational		
No education	173	2.13
Primary education	342	4.21
Secondary education	2228	27.44
Higher education	5379	66.23
Marital status		
Currently married	7706	94.87
Currently not married	416	5.13
Type of birth		
Single	7924	97.56
Multiple	198	2.44
Birth order		
1–2 order	2749	33.85
3–4 order	2209	27.20
> = 5 order	3164	38.95
Anemia		
Not anemic	5640	70.51
Mild anemia	1775	22.19
Moderate anemia	485	6.06
Severe anemia	100	1.24
No of U-5 children		
1 child	3001	37.25
2 children	3896	48.35
> = children	1161	14.40
Family size		
< 4 families	2038	27.11
> = 4 families	5479	72.89
Wealth index		
Richest	1140	14.15
Richer	1457	18.08
Middle	1774	22.02
Poorer	1880	23.33
Poorest	1806	22.41
Household head sex		
Male	6979	86.63
Female	1078	13.37
Father’s education		
No education	3579	46.84
Primary education	3180	41.62
Secondary education	584	7.64
Higher education	298	3.90
Source of drinking water		
Improved water source	4604	57.15
Unimproved water source	3453	42.85
Type of toilet facility		
Improved water source	765	9.49
Unimproved water source	7292	90.51
Residence		
Urban	873	10.84
Rural	7184	89.16
Region		
Urban	230	2.85
Rural	7302	90.62
Developing	526	6.52
Community level education		
Illiterate	5268	65.96
Literate	2718	34.04
Community poverty rate		
In poverty	3367	41.79
Above poverty	4690	58.21

### Prevalence of severe stunting among under-five children

The result of this study shows that about 18% of the children were severely stunted. Majority of severely stunted children were found in Amhara region whereas, less severely stunted children was identified in Gambella region (Table 2).

**Table 2:** The distribution of severe childhood stunting across the regions of Ethiopia, 2016

Region	Children nutritional status			Total
	Not stunted	Moderately stunted	Severely stunted	
Tigray	324	153	80	557
Afar	44	16	19	79
Amhara	782	465	344	1591
Oromia	2140	716	621	3477
Somalia	247	54	45	346
Benishangul –Gumuz	46	19	18	83
SNNPR	980	332	364	1676
Gambella	14	3	2	18
Harari	11	4	2	17
Dire Dawa	18	8	6	32
Addis Ababa	152	24	5	181
Total	4758	1794	1506	8058

### Factors associated with severe childhood stunting

The expected odds of being moderately stunted as compared to not stunted, or severe stunted compared to moderately stunted, are 1.26 (AOR = 1.26,95% CI: 1.09–1.46) times greater among those who are males than those who are female, holding the other predictors constant and keeping them in the same enumeration area.

Adjusting for other predictors and holding clusters effect, children of age group 12–23 months, 24–35 months,36–47 months and 48–59 months have 3.72, 5.52, 5.17 and 4.11 times, the odds of being moderately stunted compared to not stunted, or severely stunted compared to moderately stunted, than do children belonging to age group 6–12 months((AOR = 3.72,95%,CI:2.59–5.32),(AOR = 5.52.95%,CI:3.95–7.71),

(AOR = 5.17, 95%, CI: 3.68–7.26) and (AOR = 4.11, 95%, CI: 2.89–5.83)) respectively.

The expected odds of being severely stunted as compared to moderately stunted or moderately stunted compared to not stunted, are some 48% (AOR = 0.52,95%,CI: 0.37–0.72) less among those children of underweight mothers compared with those children of mothers normal BMI; On the other hand Children of Over-weight mothers were 3.43 times the odds of moderately stunted than not stunted, or severely stunted than moderately stunted compared to children of mothers of normal BMI (AOR = 3.43,95%,CI: 2.21–5.33) holding the other predictors constant and keeping them in the same cluster.

The expected odds of being severely stunted as compared to moderately stunted or moderately stunted compared to not stunted, are some 62% (AOR = 0.38,95%,CI: 0.20–0.74) less among those children of mothers in higher education compared with those children of mothers in no education level.

For each one-unit increase in maternal height, there is a 5% significant reduction in the odds of being severely stunted as compared to moderately stunted, or moderately stunted compared to not stunted (AOR = 0.95,95%,CI: 0.93–0.96).

Children from 20 to 35 and 36–49 years age group roughly significantly reduced by half and 60% the odds of being severely stunted compared to moderately stunted, or moderately stunted compared to not stunted, than do children from age group 15–19 years (AOR = 0.51,95%,CI: 0.32–0.81) ad (AOR = 0.41,95%,CI: 0.24–0.71) respectively adjusted for other predictors and holding cluster effect the same.

Keeping other factors constant and hold cluster effect children from middle, poorer and poorest wealth index households were 1.84, 2.13 and 2.52 time significantly increase the odds severe stunting compared to moderately stunting, or moderately stunting compared to not stunted (AOR = 1.84,95%,CI:1.27–2.67),(AOR = 2.13,95%,CI:1.45–3.14) and (AOR = 2.52,95%,CI: 1.72–3.68)respectively.

Adjusted for other predictors and children in the same cluster, living in rural regions significantly increased odds of severe stunting compared moderately stunted, or moderately stunted compared to not stunted by 1.62 (AOR = 1.62,95%,CI: 1.14–2.30) times than living in urban regions (Table 3).

**Table 3:** Multivariable multilevel ordered logistic regression among under-5 children in Ethiopia, 2016

Stunting	Random intercept model	Community level factors	House hold and individual factors	Full model
Region				
Urban		1		1
Rural		1.76 (1.24–2.48)		1.62 (1.14–2.30)**
Developing		1.23 (0.87–1.74)		1.23 (0.86–1.76)
Residence				
Urban		1		1
Rural		1.35 (0.96–1.90)		0.78 (0.51–1.18)
Community literacy				
Literate		1		1
Illiterate		0.80 (0.66–0.957)		0.90 (0.73–1.11)
Community poverty index				
In poverty		1		1
> poverty		1.37 (1.14–1.65)		1.08 (0.86–1.36)
wealth index				
Richest			1	1
Richer			1.43 (1.03–1.97)**	1.37 (0.97–1.93)
Middle			1.80 (1.30–2.50)**	1.84 (1.27–2.67)**
Poorer			2.11 (1.51–2.95)**	2.13 (1.45–3.14)**
Poorest			2.44 (1.76–3.39)**	2.52 (1.72–3.68)**
Water source				
Improved			1	1
Un improved			0.88 (0.73–1.06)	0.87 (0.72–1.05)
Toilet				
Improved			1	1
Un improved			1.12 (0.78–1.613)	1.11 (0.76–1.61)
Maternal age				
15–19			1	1
20–35			0.54 (0.34–0.85)	0.51 (0.32–0.81)**
36–49			0.48 (0.29–0.80)	0.41 (0.24–0.71)**
Maternal height			0.95 (0.93–0.96)	0.95 (0.93–0.96)**
Maternal Education				
No-education			1	1
Primary			0.80 (0.67–0.950)*	0.84 (0.67–1.02)
Secondary			0.62 (0.42–0.92)*	0.68 (0.45–1.02)
Higher			0.35 (0.19–0.66)**	0.38 (0.20–0.74)**
Maternal BMI				
Normal			1	1
Under-weight			0.49 (0.35–0.69)	0.52 (0.37–0.72)**
Over-weight			3.33 (2.15–5.17)	3.43 (2.21–5.33)**
Child age (months)				
6–12			1	1
12–23			3.64 (2.55–5.21)	3.72 (2.59–5.32)**
24–35			5.43 (3.90–7.56)	5.52 (3.95–7.71)**
36–47			5.12 (3.65–7.16)	5.17 (3.68–7.26)**
48–59			3.98 (2.82–5.60)	4.11 (2.89–5.83)**
Child sex				
Female			1	1
Male			1.28 (1.10–1.49)	1.26 (1.09–1.46)**
Birth order				
1 to 2			1	1
3 to 4			0.96 (0.77–1.18)	0.94 (0.76–1.16)
5 and above			1.24 (0.98–1.55)	1.20 (0.96–1.51)
Cut points				
Cut point 1	1.66 (1.51–1.83)	1.30 (1.00–1.60)	−6.65(−8.98,-4.31)	−6.42(−8.78,-4.07)
Cut point 2	1.71 (1.59–1.82)	1.50 (2.19–2.80)	−5.34(−7.66,-3.01)	−5.12(−7.46,-2.78)
Cluster variance(θ)	0.45 (0.34–0.60)	0.38 (0.29–0.50)**	0.41 (0.31–0.53)**	0.40 (0.30–0.52)**
Median OR	1.90 (1.75–2.09)	1.80 (1.67–1.96)	1.84 (1.70–2.01)	1.83 (1.69–2.00)**
ICC (VPC)	0.12 (0.09–0.15)**	0.10 (0.08–0.13)**	0 .11 (0.09–0.14)**	0.11 (0.08–0.14)**
PCV		15.56%	8.89%	11.11%

Note: * indicates statistical significance less than 0.05

** indicates statistical significance less than 0.01

Comparing children in clusters with high risk of stunting to children in clusters with low risk of stunting keeping other predictors effect similar the chance of stunting in high risk clusters increased by median odds ratio(MOR = 1.83,95%,CI:1.69–2.00) which correspond with 11% ICC and PCV.

## Discussion

The present study examines the magnitude and factors associated with stunting among children aged 6–59 months in Ethiopia. The independent factors those associated with severe stunting in this study were: sex of the child, child age, maternal BMI, maternal education, maternal height, maternal age, household wealth index and geopolitical region. The results would enable public health stakeholders to reform intervention designs to that were intended to reduce the frequency of stunting in Ethiopia.

Despite of various interventions to reduce the burden of stunting among children in Ethiopia the prevalence remain unacceptably high (17.8% severe and 20.4% moderately stunting). This finding is in line with results in Nigeria [19] and Tanzania [35]. This might be due to the countries socio- economic development and population living standard similarity. However, the finding is higher than results in Indonesia [11] and Nepal [36]. This could be due to variation in development level, feeding habits since majority of the current study participants were rural dwellers [3].

This study revealed that male children were significantly at higher risk of moderately stunting and severely stunting than their female counterpart. This is in concurrence with findings in Nigeria [19], Bure in Ethiopia [37], Tanzania [35], Indonesia [11] and China [38]. In Ethiopia an average, female children have a longer median duration of 6 months of predominant breastfeeding than male children 5.1 months [3]. This early introduction of supplementary food and fluid predispose male children for diarrhea and other infections, these obviously increase nutritional demand over reduced appetite [39]. The cumulative effect will put male children at higher risk of stunting ad severe stunting. Another justification, also the proportion of male preterm births is higher than female preterm births which could also contribute childhood stunting [40, 41].

The study result showed as the child age increased the risk of stunting increased up to age group 36–47 months and start to reduce at 48–59 months as compared to 6–12 months children. This finding is in agreement with study finding in Indonesia [11], Nigeria [42], Ruanda [14], Democratic Republic of Congo [43] and Libo Ethiopia [12]. The plausible reason could be children in this age groups are at developmental stage of pin grasp and crawling that able to them move and caught anything to add into their mouth. This also could predispose the child to contaminated materials and food products. As a result the children will be infected and which ended with stunting [28]. Whereas after 48 months children able to identify things to be inserted to mouth or not and there immunity also increased to reduce infection as well as risk of stunting. This implies that children in the period of crawling and weaning require particular attention to reduce childhood stunting and to enhance general wellbeing of children.

The present finding revealed that, household wealth index was negatively associated with stunting and severe stunting. This result was consistent with study conducted in North Shewa, Oromia Regional State, Ethiopia [13], Indonesia [11], Nigeria [42], Democratic Republic of Congo [43], Ruanda [14] and Nigeria [42]. This may be attributed to the fact that with increased household wealth food securities will be guaranteed to be well nutrition and maintaining age appropriate growth achievement and families in wealthiest household may be educated to care for children. Furthermore, children from wealthy household less prone to infection due to well nutrition, have access to get medical attention timely if they caught infection that reduce the probability of stunting. Therefore, to improve child health, establishing appropriately functioning economic and financial structures which supports children from disadvantaged households is required in order to improve food security and access to basic health care services.

Our findings demonstrate a reduction in risk of stunting among the children of mothers with long heights. Our findings in so doing corroborate with previous studies showing similar results Rwanda [43] and Democratic Republic of Congo [14]. The explanation to this finding might be landed on the longer mothers may come from either genetically longer families or food secured households, these made them free from short stature that resulted in birth of small newborns later became stunted [44]. It implies investment on early maternal nutrition and health is vital on childhood stunting.

Our finding demonstrated that children who had mothers whose age was above 20 years at delivery were less likely to suffer from stunting than children with adolescent mothers. Similar result reported from Democratic Republic of Congo [14] and five low income countries [43]. Adolescents are at higher nutritional demands for their growth [45]. Pregnancies at the age of this period create nutritional contention between the mother and their creature in womb. Finally this lead to in poor child growth includes stunning.

Mother’s educational status was found to be significantly negatively associated with childhood stunting. Again, this result is corroborating with previous studies, maternal education has a positive outcome in reducing the severe child stunting [14–16, 18]. One possible explanation is that knowledge that mothers get from their formal education could capable them to practice nutritional and other related behaviors that prevent chronic malnutrition/stunting. Plus to this, educated mothers have greater health seeking behavior for childhood illnesses as compared to uneducated mothers [46].

The administrative region in which the child resides has a share to play in the probability of children to being stunted and severely stunted. It was detected that children that reside in the developing and rural region of the country display a greater tendency to being stunted when compared with children in the urban regions of the country. This finding was supported with other finding in Nigeria [19, 42] This finding may be accredited to the socio-economic and education disparities and access to basic Healthcare facilities. Therefore, context based interventions particularly for developing and rural regions are mandatory in the battle of childhood stunting and better health.

In addition to fixed effect model the random effect also contributed in the determination of stunting. After accounting for predictor factors the median increase in the odds of severe stunting as compared to children at a cluster with higher risk of severe stunting to children at a cluster with lower risk of severe stunting was 83% increased. These imply significant disparity of the problem which requires a context based interventions to tackle this cyclic inter-generation linked health problem.

This study was based on a representative national data which was collected across all the regions of Ethiopia with recognized quality and representativeness at urban rural, regional as well as national level. This makes our finding to be generalized to the whole Ethiopian children and related developing countries children. However, our study was cross sectional in nature to establish causality and since the data is secondary some factors like father’s height and household food security scores could not be ruled out.

This study is significantly important for public health intervention planning and recognizing the underlying factors linked with stunting and severe stunting so as to contribute in the appropriate allocation of resources and healthcare services. Furthermore, it will help the Ethiopian government in designing and applying appropriate nutrition programs intended at improving maternal and child nutrition at both the individual and community levels most especially in developing and rural regions.

### Strength and limitation

The strengths of this study were that it used a nationally representative-survey data. Moreover, the analysis was done based on the nature of the outcome variable that has ordered categories so that ordinal logistic regression model was used and employed appropriate statistical adjustments for the cluster sampling design in the analysis. On the other hand, the limitation of this study was that we could not establish the cause and effect relationships; because of the cross-sectional nature of the study design. In addition, Variables like Dietary diversity score (DDS), household food security score (HHFSS) were not included in this analysis due to the survey did not incorporate these measures to the instrument.

## Conclusion

The current study has highlighted that the burden of chronic malnutrition was unacceptable high in Ethiopia with contextual variation. It also demonstrate the independent associated factors with stunting ad severe stunting as child age, sex, maternal height, age, education and household wealth index as well as administrative regions. Our findings designate the necessity of interventions at the individual, household and community levels. At the individual level, particular attention should be placed on child age appropriate feeding particularly young mothers regarding health and child feeding practices and maternal education as well as improving household wealth improvement. At a community level it is mandatory empower women particularly in rural and developing regions through accessing social organizations like education, healthcare services. Researchers also better to address possible cofounder variables those were not included in this study and the effect of maternal BMI on childhood growth and development.

## Data Availability

All relevant data are in the manuscript. However, the minimal data underlying all the findings in the manuscript will be available upon request.

## Abbreviations

CDC: United States Center for Disease Control and Prevention
DHS: Demographic and Health Survey
EA: Enumeration Area
EPHI: Ethiopian Public Health Institute
ICF: *International* Classification of Functioning, Disability and Health
IQR: Inter Quartile Range
HAZ: Height for age Z-score
NRERC: National Research Ethics Review Committee
SD: Standard Deviation
UNICEF: United Nations International Children’s Education Fund
PHC: Population and Housing Census
WHO: World Health Organization
